# Determination of Cell Viability—Acridine Orange/Propidium Iodide (AO/PI) Staining Method

**DOI:** 10.1111/cpr.70199

**Published:** 2026-04-22

**Authors:** Lei Wang, Bo‐qiang Fu, Ai‐jin Ma, Shuai‐shuai Niu, Yong Zhang, Ran‐ran Xu, Shi‐hui Ma, Jia‐ni Cao, Hong‐ling Zhao, Wan‐ying Wang, Hai‐ying Wang, Juan Ma, Jun Wei, Jun‐ying Yu, Qi‐yuan Li, Li‐jun Zhu, Tao Na, Jia‐le Cai, Xiao Fan, Yao‐jin Peng, Xiao‐you Yu, Qu‐bo Chen, Pei‐jun Zhai, Shi‐jun Hu, Andy‐Peng Xiang, Guo‐qiang Hua, Jia‐xi Zhou, Tong‐biao Zhao, Bao‐yang Hu, Jie Hao

**Affiliations:** ^1^ National Stem Cell Resource Center, Institute of Zoology Chinese Academy of Sciences Beijing China; ^2^ Institute for Stem Cell and Regeneration Chinese Academy of Sciences Beijing China; ^3^ Standard Committee, Chinese Society for Cell Biology Shanghai China; ^4^ Beijing Institute for Stem Cell and Regenerative Medicine Beijing China; ^5^ National Institute of Metrology Beijing China; ^6^ Beijing Technology and Business University Beijing China; ^7^ Shenzhen HHLIFE co., Ltd. Shenzhen China; ^8^ Zephyrm Biotechnologies co., Ltd. Beijing China; ^9^ Institute of Hematology & Blood Diseases Hospital Chinese Academy of Medical Sciences & Peking Union Medical College Tianjin China; ^10^ Nuwacell Biotechnology co., Ltd Hefei City Anhui Province China; ^11^ Shenzhen Medical Academy of Research and Translation Shenzhen China; ^12^ Institute of Science and Technology Information of China Beijing China; ^13^ National Institutes for Food and Drug Control Beijing China; ^14^ University of Chinese Academy of Sciences Beijing China; ^15^ The Second Clinical College of Guangzhou University of Chinese Medicine Guangzhou China; ^16^ China National Accreditation Service for Conformity Assessment (CNAS), Beijing China; ^17^ Soochow University Jiangsu Province China; ^18^ Center for Stem Cell Biology and Tissue Engineering, Key Laboratory for Stem Cells and Tissue Engineering, Ministry of Education, Sun Yat‐Sen University Guangzhou China; ^19^ Fudan University Shanghai, Cancer Center Shanghai China

‘Determination of Cell Viability—Acridine Orange/Propidium Iodide (AO/PI) Staining Method’ is the latest developed guidelines for determination of cell viability in China, jointly drafted and agreed upon by experts from the Standards Committee of Chinese Society for Cell Biology. This document specifies the method for determining cell viability using the AO/PI staining method, including principles, reagents, materials, instruments and equipment used, sample preparation methods, experimental protocols, result calculation methods, validation methods and results presentation. This standard is applicable for the viability testing of nucleated mammalian cells, and the determination of viability of other nucleated animal cells may be carried out by reference to this standard. This standard is not applicable for the testing of viability of plant cells and enucleated cells. This standard was originally released by the China Society for Cell Biology on 28 October 2024. We hope that this standard will serve as a valuable resource for scientific research, enhance the quality control of cellular therapeutic products, and facilitate the monitoring and release of cells in cell banks worldwide.

## 1 Scope

This document specifies the method for determining cell viability using the AO/PI staining method. It includes principles, reagents, materials, instruments and equipment used, methods of sample preparation, experimental protocols, result calculation methods, validation methods and results presentation. This document is applicable for assessing the viability of nucleated mammalian cells and may also serve as a reference for testing the viability of other nucleated animal cells.

This document is not applicable to testing the viability of plant cells and enucleated cells.

## 2 Normative References

There are no normative references in this document.

## 3 Terms and Definitions, Acronyms

### 3.1 Terms and Definitions

#### 3.1.1 Cell Viability

The proportion of live cells in the total cell population.

#### 3.1.2 Single Cell Suspension

A single‐cell suspension without cell clumps.

### 3.2 Acronyms

AO: acridine orange.

PI: propidium iodide.

## 4 Principle

The AO/PI staining solution consists of a double‐stranded DNA‐binding dye AO that emits green fluorescence and a double‐stranded DNA‐binding dye PI that emits red fluorescence. AO can pass through intact cell membranes and enter the nuclei of all cells (both live and dead), where it emits green fluorescence under the excitation of 480/30 nm light. PI, on the other hand, can only pass through damaged cell membranes and enter the nuclei of dead cells, where it emits red fluorescence upon excitation of 525/30 nm light. In dead cells, when AO and PI coexist, fluorescence resonance energy transfer (FRET) occurs at the appropriate ratio of AO to PI, causing the green fluorescence of AO to be quenched and the red fluorescence of PI to be enhanced, resulting in dead cells emitting red fluorescence. Using a fluorescence cell counter, the ratio of live cells can be determined by counting the number of live and dead cells, which is also referred to as cell viability.

## 5 Reagents or Materials

5.1 The haemocytometer should be specified for use with the fluorescence cell counter.

5.2 The pore size of cell filter should be between 40 to 100 μm.

5.3 The cell diluent's osmotic pressure and pH value shall be compatible with the physiological state of the tested cells.

5.4 For the AO/PI staining solution, the AO and PI shall be in an appropriate ratio to meet the requirements for cell staining and detection.

## 6 Instruments and Equipment

6.1 Fluorescence cell counter: It shall have excitation light sources capable of exciting AO at 480/30 nm and PI at 525/30 nm, along with corresponding fluorescence detection channels, which shall be calibrated.

6.2 Micropipette: After calibration, it meets the testing requirements.

6.3 Centrifuge: The centrifugal force required for the cell suspension should meet the specified requirements, with the upper limit of centrifugal force being ≥ 1000×g.

## 7 Sample

7.1 The cells shall be suspended in a single, dispersed state.

7.2 The sample volume shall comply with the loading volume requirements of the fluorescence cell counter.

7.3 The cell concentration should range from 1 × 10^5^ to 1 × 10^7^ cells/mL.

## 8 Test Procedure

Warning: AO and PI are irritants to the human body. Handle with care and take appropriate precautions to prevent direct contact or inhalation.

### 8.1 Preparation of Cell Suspension

#### 8.1.1 Cell Suspension With Calibrated Cell Concentration

If the cell concentration is within the required range, take an appropriate amount of cell suspension (suspension A).

If the cell concentration exceeds the specified maximum, take an appropriate amount of cell suspension, add an appropriate amount of cell diluent, and resuspend the cells. Dilute the single‐cell suspension to a concentration of 1 × 10^6^/mL (suspension A).

If the cell concentration is lower than the specified minimum, transfer the cell suspension to a 15 mL centrifuge tube and centrifuge at (150 ~ 400) × g for 5 min. Discard the supernatant, and based on the indicated cell concentration, add an appropriate amount of cell diluent to resuspend the cell pellet, creating a single‐cell suspension (suspension A) with a concentration of 1 × 10^6^ cells/mL.

Note: For special cell types, if adjusting the centrifugation parameters is necessary, optimisation should be performed.

#### 8.1.2 Uncalibrated Cell Suspension

Take a small aliquot of the cell suspension and add it to a haemocytometer. Perform a total cell count using a fluorescence cell counter.

Consider specifying the method or equipment used for counting, if relevant.

If the cell concentration is within the required range, take an appropriate amount of cell suspension (suspension A).

If the cell concentration exceeds the specified maximum concentration or is lower than the specified minimum concentration, proceed according to Section 8.1.1.

### 8.2 Sample Preparation

For each sample group, prepare three parallel samples (suspension A). Add AO/PI dye in appropriate proportion to suspension A and mix well, then make suspension B.

### 8.3 Adding Samples

According to the volume requirements of the fluorescence cell counter, take an appropriate volume of suspension B, add it to a haemocytometer, let it stand at room temperature in the dark, and then perform the test within 10 min. Measure three replicates.

### 8.4 Detection

Place an instrument‐specific haemocytometer into the fluorescence cell counter, select the appropriate fluorescence mode and measure the number of green and red fluorescent cells with the instrument's automatic autofocus feature.

## 9 Result Calculation

Cell viability is calculated according to Equation (1):

(1) $$\chi =\frac{N_{h}}{N_{s}+N_{h}}$$

In the formula:

χ‐cell viability;

*N*_*h*_‐number of live cells exhibiting green fluorescence;

*N*_*s*_‐number of dead cells exhibiting red fluorescence.

The final result is represented by the arithmetic mean of three independent measurement results obtained under repeated conditions, with three significant figures retained. The mean is calculated according to Equation (2) and is expressed in units of (%).

(2) $$\bar{x}=\frac{\sum_{i}^{n}x_{i}}{n}$$

In the formula:

$\bar{\bar{x}}$‐the average cell viability is expressed as a percentage (%);

$x_{i}$‐the cell viability of each replicate sample is expressed as a percentage (%);

*n*‐represents the number of replicate samples.

## 10 Precision

The absolute difference between three independent measurement results obtained under identical experimental conditions should not exceed 10% of the arithmetic mean.

## 11 Test Records and Results Reports

Record experimental conditions, measurement results, and calculation results. Refer to Appendix A for the record template.

The report should include the test subjects, adopted standards, methodology, results, any observed anomalies, and the date.

## Author Contributions

Jie Hao, Bao‐yang Hu, and Tong‐biao Zhao contributed to conception and design. Lei Wang, Bo‐qiang Fu, Ai‐jin Ma, Shuai‐shuai Niu drafted and revised the manuscript. Lei Wang, Ran‐ran Xu, Shi‐hui Ma, Wan‐ying Wang, Hai‐ying Wang, Juan Ma, Jia‐le Cai, and Xiao Fan performed the validation experiment. Yong Zhang, Jia‐ni Cao, Hong‐ling Zhao, Jun Wei, Jun‐ying Yu, Qi‐yuan Li, Li‐jun Zhu, Tao Na, Yao‐jin Peng, Xiao‐you Yu, Qu‐bo Chen, Pei‐jun Zhai, Shi‐jun Hu, Andy‐Peng Xiang, Guo‐qiang Hua, and Jia‐xi Zhou critically read and revised the manuscript.

## Funding

This research is supported by the Strategic Priority Research Programme of the Chinese Academy of Sciences, Grant No. XDB1030000 and XDC0200000, and National Key Research and Development Programme of China, Grant No. 2024YFA1106900, 2021YFA1101604 and 2024YFA1108302, Chongqing Municipal Major Project for Technological Innovation and Applied Development, Grant No. CSTB2025TIAD‐STX0044.

## Data Availability

Data sharing not applicable to this article as no datasets were generated or analysed during the current study.

## Appendix A

(Informative)

Cell viability determination AO/PI staining method detection record.

Test date:_______ Year _______ Month _______ Day Test basis: _______

**Table:**

Sample information					
Name	Lot NO.			Specification	Consumption
			
			
Material					
Name	Lot NO.			Expiration date	Manufacturer
AO/PI					
Sample dilution					
Detection equipment					
Name	Model			Serial NO.	Manufacturer
Fluorescence cell counter					
Determination method					
Preparation of Test Sample Solution: Take the test sample cells, mix them gently, aspirate ______ μL of the cell suspension, add it to ______ μL of the sample diluent, dilute it by ______ times, and gently pipette to mix evenly. Prepare three tubes in parallel according to this method. Staining: Take ______ μL of well—mixed test sample solution from each tube, mix each with an equal volume of AO/PI evenly, and then aspirate ______ μL of each mixture and add it to the wells of the haemocytometer, with a total of three wells. Detection and Calculation: Insert the haemocytometer into the fluorescence cell counter, use the “AO/PI Cell Viability” module for detection, set the dilution ratio as 1:1, read and record the viable cell density and cell viability, and calculate the average value and RSD value of the measurement results of the three wells.					
**Result**					
Cell viability (%)	1	2	3	Average	RSD Value (%) < 10%
					□ Y □ N
**Conclusion:**____________________ **Note:**					
Tester/Date____________________			Reviewer/Date__________________		

